# Survival and Growth of *Orientia tsutsugamushi* in Conventional Hemocultures

**DOI:** 10.3201/eid2208.151259

**Published:** 2016-08

**Authors:** Sabine Dittrich, Elizabeth Card, Weerawat Phuklia, Williams E. Rudgard, Joy Silousok, Phonelavanh Phoumin, Latsaniphone Bouthasavong, Sarah Azarian, Viengmon Davong, David A.B. Dance, Manivanh Vongsouvath, Rattanaphone Phetsouvanh, Paul N. Newton

**Affiliations:** Mahosot Hospital, Vientiane, Laos (S. Dittrich, E. Card, W. Phuklia, W.E. Rudgard, J. Silousok, P. Phoumin, L. Bouthasavong, S. Azarian, V. Davong, D.A.B. Dance, M. Vongsouvath, R. Phetsouvanh, P.N. Newton);; Centre for Tropical Medicine and Global Health–University of Oxford, Oxford, United Kingdom (S. Dittrick, D.A.B. Dance, R. Phetsouvanh, P.N. Newton);; London School of Hygiene and Tropical Medicine, London, United Kingdom (W.E. Rudgard, S. Azarian, P.N. Newton)

**Keywords:** Orientia tsutsugamushi, hemoculture, blood culture, Rickettsia spp., culture, molecular diagnostic, Lao, Laos, scrub typhus, diagnostic, bacteria

## Abstract

*Orientia tsutsugamushi,* which requires specialized facilities for culture, is a substantial cause of disease in Asia. We demonstrate that *O. tsutsugamushi* numbers increased for up to 5 days in conventional hemocultures. Performing such a culture step before molecular testing could increase the sensitivity of *O. tsutsugamushi* molecular diagnosis.

*Orientia tsutsugamushi*, the causative agent of scrub typhus, has long been a pathogen of major public health concern in the Asia-Pacific region (*1*,*2*). Reports from India, China, and Southeast Asia suggest that a substantial proportion of fevers and central nervous system infections are caused by this bacterium (*3*,*4*). The World Health Organization has called scrub typhus “probably one of the most underdiagnosed and underreported febrile illnesses requiring hospitalization in the region” (*5*). Furthermore, *Orientia-*infected patients are emerging outside the so-called tsutsugamushi triangle, from the Middle East, Africa, and South America (*6*–*8*). *Orientia* spp. DNA was recently detected in rodents from Asia, Europe, and Africa (*9*). 

To improve patient management and clarify the epidemiology and pathogenicity of *O. tsutsugamushi*, physicians and researchers need sensitive and specific diagnostic tools (*10*). The current diagnostic reference standard is a 4-fold antibody rise between acute- and convalescent-phase serum samples; however, because results are determined retrospectively, they cannot influence patient management. *O. tsutsugamushi* is an intracellular pathogen*,* and traditionally its growth has been assumed to require specialized cell culture at Biosafety Level 3, which is only available at a limited number of specialized centers. Therefore, molecular detection of *O. tsutsugamushi* in patients’ EDTA-blood buffy coat has become the tool of choice for routine diagnosis and epidemiologic studies (*11*,*12*). In other pathogens with low bacterial loads, propagation of the organism before molecular amplification has increased target density and improved sensitivity of diagnostic tools such as quantitative PCR (qPCR) or isolation in cell cultures (*13*). In line with these findings, we hypothesized that *O. tsutsugamushi* can survive and potentially grow in conventional hemoculture media within the co-inoculated human host cells and that this capacity for growth could be used to improve diagnostic and analytical sensitivities.

## The Study

We conducted this study at the Microbiology Laboratory, Mahosot Hospital, Vientiane, Laos, the only microbiology laboratory in Vientiane with a routine, accessible hemoculture service for sepsis diagnosis (*4*,*14*). Study patients provided written informed consent; ethical approval was granted by the Oxford Tropical Research Ethics Committee, University of Oxford (Oxford, UK), and the National Ethics Committee for Health Research, Laos.

One pair of hemocultures was incubated aerobically at 35°C–37°C for 7 days. As part of a weekly routine molecular diagnostic service, admission EDTA anti-coagulated buffy coats (≈200 μL) from febrile patients were collected to test for *O. tsutsugamushi* and *Rickettsia* spp. by qPCR (*11*). Serial dilutions of plasmids (pGEM-T Vector Systems; Promega, Southhampton, UK) and nontemplate controls were included in all runs as external controls; they always showed the appropriate result. During the 2014 rainy season (May–October), we collected consecutive hemoculture fluids after 24 h of incubation (0.5 mL/bottle; total 1 mL/patient; n = 760) (*15*). Each aliquot contained ≈0.1 mL blood or 0.01 mL buffy coat per mL media. If the EDTA buffy coat qPCR result (cycle quantitation value≤40) was positive within the 7-day hemoculture incubation period (n = 11), the respective bottles were sampled at subsequent times so PCR positivity could be estimated (Table). 

**Table:** Results of testing HCF from 21 patients whose samples had tested positive for *Or*i*entia tsutsugamushi* by qPCR on EDTA buffy coat after >1 days of incubation, Laos, 2014*

Patient code	Day of incubation of HCF†				
1	2	3	5	7
29931	+ (Cq ≈34)				
30009	–				
30029	–				
30100	– (Cq ≈42)		–	+ (Cq ≈35)	+ (Cq ≈38)
30104	+ (Cq ≈38)				
30316	+ (Cq ≈37)		–		+ (Cq ≈38)
30329		+ (Cq≈39)		+ (Cq ≈35)	+ (Cq ≈37)
30379	+ (Cq ≈34)				
30416	+ (Cq ≈35)		+ (Cq≈40)	+ (Cq ≈40)	
30442	–				–
30446	+ (Cq ≈37)				–
30581	–		+ (Cq≈32)	+ (Cq ≈30)	+ (Cq ≈34)
30600	–				+ (Cq ≈32)
30862	+ (Cq ≈33)				
30874	+ (Cq ≈30)				
30920					
31120	+ (Cq ≈31)				
31199	+ (Cq ≈30)				
31209	+ (Cq ≈31)				+ (Cq ≈21)
31230	+ (Cq ≈31)			+ (Cq ≈31)	+ (Cq ≈32)
31231	+ (Cq ≈32)			+ (Cq ≈30)	+ (Cq ≈37)

*For each patient, 2 hemocultures bottles were used (Pharmaceutical Factory No. 2, Vientiane, Laos): 1.7 g tryptic hydrolysate casein, 0.3 g soy peptone, 0.5 g sodium chloride, 0.25 g potassium phosphate, 0.25 g dextrose, 0.025 g sodium polyanetholsulfonate/100 mL) were inoculated with blood in a 1:10 dilution. Cq, cycle quantitation; HCF, hemoculture fluids; qPCR, quantitative PCR; –, sample available/negative; +, sample available/positive; blank cell, no sample available.  †Cq values are noted in parentheses to illustrate the increase in template.

Hemoculture fluids were centrifuged (10 min at 15,900 × *g*) to pellet intracellular and extracellular bacteria, and DNA was extracted by using a method that removes inhibitors with benzyl alcohol (guanidine hydrochloride lysis/column extraction) (*16*). The same qPCR protocols were used for buffy coat (1 μL/reaction) and hemoculture fluids (7 µL/reaction), with bovine serum albumin (40 μg/reaction) added in the latter (*11*,*13*). We estimated bacterial loads using plasmid standard curves and calculated bacterial multiplication factor by dividing bacterial numbers, at follow-up days, by the number in the first sample.

Of the 760 buffy coat samples, 21 were positive for *O. tsutsugamushi* by qPCR; therefore, to estimate survival and growth, we collected aliquots of hemoculture fluids during days 1–7 (n = 11). During that period, 100/760 (13%) hemocultures were positive for other organisms; however, none of the known *O. tsutsugamushi*–infected patients were co-infected with an alternative pathogen. In addition, hemoculture fluids from the first 277 patients with buffy coats negative for *O. tsutsugamushi* by qPCR*,* underwent *O. tsutsugamushi* qPCR testing to explore whether using hemoculture fluids as an additional diagnostic sample could improve molecular detection of *O. tsutsugamushi*.

For nearly all (20/21) of the *O. tsutsugamushi–*positive patients, >1 hemoculture samples were available. Of these, >1 hemoculture sample from 17/20 (85%) patients tested positive (Table). Despite the initial 1:10 dilution of blood in hemoculture fluid, 66% (14/21) of patients with buffy coat–positive qPCR results also had a positive result in the first hemoculture fluid sample (Table). Due to logistical constraints, only 11/21 patients could be tested at multiple time points; samples from 7/11 (63%) showed a marked increase in *O. tsutsugamushi* density between day 1 and day 7 (Figure). The increase ranged from 2-fold to 210-fold (median 3.25-fold), and data suggest that *Orientia* bacterial density peaked in the first 5 days and subsequently declined (Figure).

**Figure:**
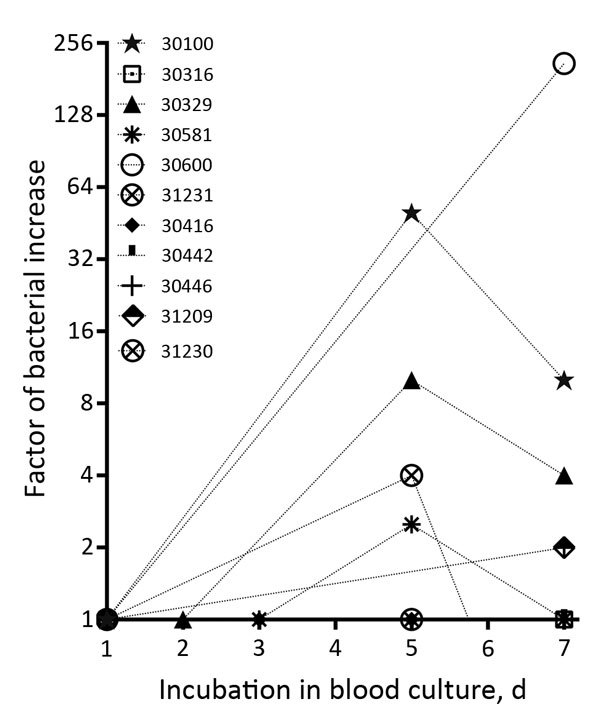
Growth curve of *Or*i*entia tsutsugamushi* in hemoculture bottles from individual patients, Ventiane, Laos, 2014. The increase in bacterial numbers is represented as bacterial multiplication factor and plotted on a log_2_ axis. Patient codes in key match those listed in the Table.

During the same period, 10/760 patients were diagnosed with *Rickettsia* spp. infection by blood culture qPCR. In contrast to the positive results for *O. tsutsugamushi*, all samples were negative by *Rickettsia* spp. hemoculture fluid qPCR, regardless of sampling day.

Of samples from the 277 patients tested for *O. tsutsugamushi* in hemoculture fluid incubated for 24 h, those from an additional 3 patients, negative by qPCR at admission, were positive (cycle quantitation value ≤40). In this pilot study, combining buffy coat and hemoculture fluid testing, the number of *O. tsutsugamushi*–positive patients increased by 3 (27%). Results are consistent with our hypothesis that qPCR on incubated hemoculture fluid alone or in combination with admission buffy coat testing could improve the sensitivity of molecular diagnosis of *O. tsutsugamushi* infection.

## Conclusions

Our findings suggest that *O. tsutsugamushi* remains viable and growing in hemoculture, presumably in human leukocytes. This observation opens up new possibilities for detecting *O. tsutsugamushi* and other intracellular organisms, albeit seemingly not *Rickettsia* spp. With the discovery of *Orientia* spp. outside the tsutsugamushi triangle, improved tools are needed to aid the timely identification of infections in disease-endemic communities and among returning travelers to facilitate appropriate treatment (*4*). In areas without sophisticated laboratory facilities, the combination of hemoculture amplification and direct diagnostic (e.g., antigen capture) has been proposed for simplified *Salmonella enterica* serovar Typhi diagnosis and resistance molecular screening (*13*). Similarly, innovative approaches to identify *O. tsutsugamushi* infections after >1 days could be developed. Because *O. tsutsugamushi* is categorized as a Hazard Group 3 pathogen, sampling and processing of hemocultures from patients with suspected scrub typhus should be conducted with appropriate biosafety precautions.

Furthermore, the possibility of using hemoculture as a simple transport tool for samples from remote areas should be investigated. Clinical isolates from remote areas can also show *O. tsutsugamushi* diversity, which can guide the development of diagnostic tests and vaccines (*3*).

The underlying mechanisms and the reason *O. tsutsugamushi*, but not *Rickettsia* spp., grows in hemoculture fluid are unclear. We compared the diagnostic possibilities of preincubating blood samples in hemoculture fluid before qPCR with using qPCR on buffy coat samples to investigate its advantage as a diagnostic tool. Further investigations are needed to confirm our findings, explore whether *O. tsutsugamushi* really multiplies in hemoculture fluids, and assess multiplication rates of host cells and pathogens in these fluids. Such studies can provide evidence for the optimal system and number of incubation days that would maximize the effects for the *O. tsutsugamushi* diagnostic. These findings could also affect the detection of other underdiagnosed intracellular pathogens that scientists assume cannot be detected in hemoculture fluids.
